# Atmospheric Correction of Satellite GF-1/WFV Imagery and Quantitative Estimation of Suspended Particulate Matter in the Yangtze Estuary

**DOI:** 10.3390/s16121997

**Published:** 2016-11-25

**Authors:** Pei Shang, Fang Shen

**Affiliations:** State Key Laboratory of Estuarine and Coastal Research, East China Normal University, Shanghai 200062, China; 52142601008@ecnu.cn

**Keywords:** atmospheric correction, GF-1/WFV, Landsat-8/OLI, Suspended Particulate Matter

## Abstract

The Multispectral Wide Field of View (WFV) camera on the Chinese GF-1 satellite, launched in 2013, has advantages of high spatial resolution (16 m), short revisit period (4 days) and wide scene swath (800 km) compared to the Landsat-8/OLI, which make it an ideal means of monitoring spatial-temporal changes of Suspended Particulate Matter (SPM) in large estuaries like the Yangtze Estuary. However, a lack of proper atmospheric correction methods has limited its application in water quality assessment. We propose an atmospheric correction method based on a look up table coupled by the atmosphere radiative transfer model (6S) and the water semi-empirical radiative transfer (SERT) model for inversion of water-leaving reflectance from GF-1 top-of-atmosphere radiance, and then retrieving SPM concentration from water-leaving radiance reflectance of the Yangtze Estuary and its adjacent sea. Results are validated by the Landsat-8/OLI imagery together with autonomous fixed station data, and influences of human activities (e.g., waterway construction and shipping) on SPM distribution are analyzed.

## 1. Introduction

Owing to terrestrial sediment inputs and bottom sediment resuspension, coastal and estuarine waters are often quite turbid. Suspended particulate matter (SPM) not only plays an important role in estuarine and coastal geomorphology evolution, pollutant transport, water quality changes, but is also an important factor to be considered in harbor and navigation channel construction [1,2,3]. Having the advantages of observing large area synchronously, timely, and economically, remote sensing technique makes dynamic SPM monitoring possible. As a trade-off between launch costs, signal-to-noise ratio, bands setting and other aspects, traditional ocean color sensors usually have a coarse spatial resolution (250 to 100 m pixel size), which is insufficient for water quality assessment of estuarine and coastal waters [4]. Thus imagery from higher resolution sensors (e.g., Landsat-8/OLI, Sentinel-2/MSI, and GF-1/WFV) with appropriate atmospheric correction methods for turbid waters, is of great interest in water quality monitoring over estuarine and coastal waters [5].

The purpose of the atmospheric correction is to remove atmospheric and surface effects from the signal measured by satellite sensors [6]. For open ocean waters, atmospheric correction method usually use two or more near-infrared (NIR) wavebands where the marine signal is assumed to be zero [7], thus the signal in the NIR can be regarded as entirely atmospheric, and is used to determine the aerosol model. In turbid waters, however, the signal in the NIR is not negligible due to high concentrations of particulate matter, and atmospheric correction for open ocean may lead to low or even negative marine reflectance in the visible bands [8]. To solve this problem, one common way is to use short-wave infrared (SWIR) wavelengths where the marine signal can be assumed to be zero even in turbid waters [9,10]. Lacking of SWIR band, this atmospheric correction method for turbid water does not apply to GF-1/WFV imagery, which have been widely used in applications like land use dynamic, geographical mapping, geological interpretation and so on [11,12,13,14]. The potential of GF-1/WFV imagery in inland and coastal water quality assessment has been gaining more and more interest thanks to its high spatial resolution (about 16 m ground pixel size), relatively short revisit period (4 days) and wide scene swath (800 km, four cameras) [15,16]. However, the lack of proper atmospheric correction methods have constrained its applications in water quality assessment over turbid waters. One possible solution is to run the atmosphere radiative transfer model like 6S and MODTRAN with input parameters about observation geometry from image metadata and aerosol properties from other supplementary data (e.g., data from ground weather stations or from matched satellite aerosol products) [16,17], one disadvantage of this method is that the supplementary data is not always available.

The objective of this work was to develop an automatic method for atmospheric correction over extremely turbid waters using the GF-1/WFV imagery. A lookup table (LUT) is created by coupling the Second Simulation of a Satellite Signal in the Solar Spectrum (6S) atmosphere radiative transfer model and the water Semi-Empirical Radiative Transfer (SERT) model. By using the lookup table, no supplementary data is needed for the atmospheric correction process, and there is no special requirement for the band set. Our method is simple in principle and can be adjusted for other sensors easily. The processing time is also acceptable, about 15 min for one scene using the MATLAB R2012b platform on a PC with a 3.40 GHz CPU and a 20.0 GB RAM. Generating the lookup table takes a while (about 5 days on the same PC) but it only has to be calculated once.

## 2. Materials

### 2.1. Study Area

**Figure 1:**
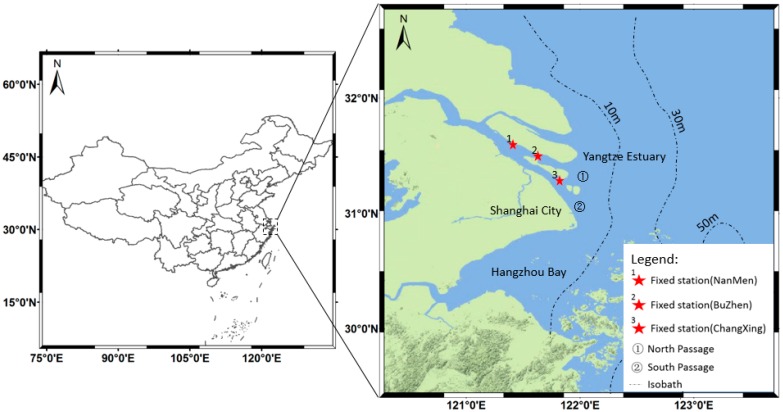
Study area of this work. Red stars indicate the locations of fixed stations.

As the largest river in Asia and the third largest river in the world, the Yangtze River discharges a huge amount of water and sediment into the East China Sea. The annual average runoff is up to 9 × 10^11^ m^3^ and sediment discharge is as high as 4 × 10^8^ t from 1951 to 2000, which are strongly affected by the dry and flood season variations [3]. The Yangtze Estuary is about 120 km long from east to west and 80 km wide from south to north (see Figure 1), rich sediment discharge and strong resuspension effect lead to a high level of SPM concentration in Yangtze Estuary all year round, making it a sediment-dominated case II waters.

The Yangtze Estuary waters can be roughly divided into four regions according to TSM concentration [18]: (1) extremely turbid waters, located 121°55′ E–122°15′ E, 31°15′ N–31°45′ N, with an average between 500 and 900 mg/L and a maximum about 2500 mg/L; (2) highly turbid waters, located 122°15′ E–122°30′ E, 31°00′ N–32°00′ N, with an average between 250 and 500 mg/L; (3) moderately turbid waters, located between 15 m and 30 m isobaths, with an average between 80 and 250 mg/L; (4) low turbidity waters, located beyond 30 m isobaths, with the concentration lower than 80 mg/L.

Yangtze Estuary also plays a crucial role in national economy field: Shanghai city, located in the south of the Yangtze Estuary, is the financial center of China; The Port of Shanghai, located in the Yangtze river delta front, has the largest container throughput in the world; The Yangtze Estuary deep-water channel, located in the Yangtze Estuary North Passage, have brought a lot of shipping benefits to Shanghai.

### 2.2. Satllite Data

On board of the GF-1 satellite there are four Wide Field of View cameras (WFV 1–4), which are four band multispectral CCD cameras with a spatial resolution of about 16 m (the ground pixel size may differ from 16 m a little due to reprojection). By mosaicking images obtained from four WFV cameras simultaneously the swath width can be as wide as 800 km, which is the widest in the world among sensors of the same spatial resolution level [19]. For this reason, the GF-1/WFV imagery has relatively short revisit period of 4 days, which increases the chance of acquiring cloud-free images.

In this study, imagery from the well-established Operational Land Imager (OLI) on the Landsat-8 platform are used for the validation. The Landsat-8/OLI is a nine-bands push broom sensor with eight bands at 30 m spatial resolution and one panchromatic band at 15 m resolution. The swath width is 185 km and revisit period is 16 days. Compared with the Enhanced Thematic Mapper Plus (ETM+) on Landsat-7, the Landsat-8/OLI have a higher signal-to-noise ratios (SNR) and a better quantization of 12 bits instead of previous 8 bits [20], making it a useful tool for monitoring coastal sediment at high resolution and high quality [5,10].

The GF-1/WFV imagery outperforms Landsat-8/OLI imagery in spatial resolution, revisit period and swath width, but is inferior to it in SNR, quantization (10 bits for GF-1/WFV) and band set [15]. Despite those shortcomings, the GF-1/WFV imagery’s quality is sufficient for TSM retrieval of inland and coastal waters [15]. A comparison of two imageries’ band range, average extraterrestrial solar irradiance *F*_0_ and SNR is shown in Table 1. The SNR value is an average of Liang’s result [15] calculated by local variance method [21] over 4 inland waters.

GF-1/WFV imagery at L1A can be obtained free of charge from the China Center for Resources Satellite Data and Application (CRESDA) website (http://www.cresda.com). The L1A product is preliminary reprojected to the UTM (N51 zone) projected coordinate system using ENVI software (version 5.1), then the top-of-atmosphere radiance (*L*_TOA_) is calculated from digital number, DN, via:

(1) $$L_{\mathrm{TOA}}=\mathrm{DN}\times \mathrm{Gains}+\mathrm{Offsets},$$

where Gains and Offsets values are provided by CRESDA absolute radiometric calibration coefficient file [22]. The Landsat-8/OLI imagery at L1T is obtained from USGS Global Visualization Viewer (http://glovis.usgs.gov/) and processed with ACOLITE (version 20160520.1), which is an atmospheric correction and processor for the Landsat-8 Operational Land Imager (OLI) and Sentinel-2A MultiSpectral Imager (MSI) developed by RBINS. It allows simple and fast processing of L8 and S2A images for marine and inland water applications [23]. The remote sensing reflectance (*R_rs_*_)_ of Landsat-8/OLI imagery at visible and NIR bands were calculated using ACOLITE with the per pixel variable epsilon SWIR atmospheric correction method [5,10]. In this study seven scenes of GF-1/WFV images and two scenes of Landsat-8/OLI images are used (listed in Table 2), including four GF-1/WFV images (two scenes of WFV1 and two scenes of WFV2) acquired on 4 November 2011 for mosaicking and two Landsat-8/OLI images acquired on the same day for cross-validation.

### 2.3. In Situ Data

The in situ datasets were collected in five cruise campaigns during 2013–2015 at the Yangtze estuary and its adjacent sea. Radiometric measurements and water samplings were carried out simultaneously at each mooring station.

The SPM concentration was determined gravimetrically by filtering the surface water samples on 0.7 μm Whatman GF/F glass fiber filter. The blank and sample-filled filters were rinsed with Milli-Q water to remove salts, dried and then reweighed on a high-precision balance in laboratory.

Radiometric measurements were recorded using a system of HyperSAS spectroradiometers (Satlantic Inc., Halifax, NS, Canada) designed for above-water measurements of ocean color. Three sensors (two radiacne sensors and one irradiance sensor) mounted to the SAS platform follow the specific observation geometry as reconmmended by the NASA protocols [24]. One radiance sensor is pointed to the sea to measure the total radiance above the water (*L*_tot_), while the other is pointed to measure the sky radiance (*L*_sky_) necessary for correction of the contribution from sky reflection. The sea and sky radiance sensors are pointed at the same nadir and zenith angles (between 30° and 50° with an optimun angle of 40°), respectivly. To minimize the sunglint effects, the sea sensor is pointed at the azmuth angle between 90° and 180° away from the solar plane, with an optimum angle of 135°. The irradiance sensor is used to measure the downwelling irradiance (*E*_d_). Therefore, the remote-sensing reflectance (*R_rs_*) is determined by the ratio of water-leaving radiance (*L*_tot_-*ρL*_sky_) and downwelling irradiance (*E*_d_), where *ρ* is the sea surface reflectance factor [25].

## 3. Methods

### 3.1. GF-1/WFV Imagery Processing

One advantage of the GF-1 satellite is that there are four WFV cameras on board, thus we can get imagery with a wide scene swath by mosaicking images acquired by different cameras at the same time. The field-of-view (FOV) of neighboring cameras overlapped by 0.44°, thus neighboring images overlapped to each other with a striped region. Because of having different viewing geometries, the same object will have different signal response in different images captured by neighboring cameras, and it will cause the chromatism between images. In order to solve this problem, we assume that the *L*_TOA_ of corresponding pixels at each band in neighboring images have a linear relationship, that is:

(2) $$L_{\mathrm{TOA}}^{1}\left(n\right)=S\left(n\right)\times L_{\mathrm{TOA}}^{2}\left(n\right)+I\left(n\right),$$

where $L_{\mathrm{TOA}}^{1}$ is the top-of-atmosphere radiance captured by one camera and $L_{\mathrm{TOA}}^{2}$ by its neighboring camera; S and I is the slope and intercept of the linear regression equation separately; n denotes the nth band of the image. This relationship can be acquired by a linear regression method using the *L*_TOA_ of corresponding pixels at each band in neighboring images’ overlap region, then the relationship is applied to the entire scene of one image to adjust its *L*_TOA_ to the reference image. In the following atmospheric correction procedure, the viewing geometry of the adjusted image is replaced by the referenced image’s.

The signal-to-noise ratio of the WFV cameras is relatively low comparing to the OLI imager (Table 1), which will cause the “noisy” phenomenon for a homogeneous water body. In order to reduce the noise and keep the spatial variability as much as possible at the same time, a 3 × 3 box sliding-window smoothing is applied to the entire scene.

### 3.2. Recalibration of SERT Model

The SERT model proposed by Shen et al. [26] is a semi-empirical radiative transfer model based on the Kubelka-Munk two-stream approximation of radiative transfer theory in water media [27]. For an optically deep (semi-infinite) turbid medium, the underwater reflectance *r* is given by [26,28]:

(3) $$r=\frac{b_{b}}{b_{b}+a+\sqrt{a\left(a+2b_{b}\right)}}=\frac{b_{b}/a}{1+b_{b}/a+\sqrt{\left(1+2b_{b}/a\right)}}$$

For sediment-dominated waters like Yangtze Estuary waters, we simply assume that the ratio *b_b_/a* is proportional to the suspended sediment concentration. As further explained, we defined the ratio *b_b_/a* as [29]:

(4) $$\frac{b_{b}}{a}=\frac{b_{bo}+{b^{*}}_{bs}\times C_{\mathrm{SPM}}}{a_{o}+{a^{*}}_{s}\times C_{\mathrm{SPM}}},$$

where *o* stands for “other”, and *s* for sediment. The assumption is strictly true if *b_bo_* = 0 and a^*^_s_ = 0. The *b_bo_* can be regarded as negligible compared with *b_b_*_s_ and the second condition would be valid if *a_o_* >> *a_s_*.

Thus, based on the aforementioned assumption, according to Equation (3), the remote sensing reflectance (*R_rs_*) can be expressed as:

(5) $$R_{rs}=\frac{\alpha \beta C_{\mathrm{SPM}}}{1+\beta C_{\mathrm{SPM}}+\sqrt{\left(1+2\beta C_{\mathrm{SPM}}\right)}}$$

where *α* and *β* are equation constants and wavelength dependent, which can be recalibrated and optimized by in situ measurements of *R_rs_* and SPM concentration (*C*_SPM_) using the non-linear regression. The constant *α* is similar to the constant f in the Gordon’s model [30], which is affected by illumination conditions and water types. While *β* is the ratio of mass-specific backscattering coefficient b_b_* and absorption coefficient *a*. The SERT model has been proved to be validate for highly turbid waters like Yangtze Estuary waters with in situ measurements and satellite products [26,27,29].

There are 228 *R_rs_*_-_*C*_SPM_ data pairs collected in cruises during 2013–2015. In order to control the data quality, in situ data pair who’s *R_rs_* at 555 nm exceeded 50% absolute percentage difference (APD) is considered to be invalid. The APD is calculated by:

(6) $$\mathrm{APD}=\left(\frac{\left|X_{i}-Y_{i}\right|}{Y_{i}}\right)\times 100\text{\%}$$

where *X* is the modelled *R_rs_* calculated by Equation (5) with in situ *C*_SPM_ and *α* *β* at 555 nm (see Table 2 in [27]); *Y* is the in situ measured *R_rs_*. The subscript *i* represents an individual sample. After filtering, 66 data pairs were left. Two reasons can explain the rejection of so many *R_rs_*-*C*_SPM_ data pairs: (1) even pay attention to, there still exist uncertainties when measuring *R_rs_* due to the variability of the illumination conditions in the field; and (2) as previously discussed, the SERT model is valid for limited types of waters (sediment-dominated waters), however, our dataset covers a wide range of water types, so the model is not applicable to data pairs collected in other types of waters and those data pairs were rejected by the filtering strategy discussed above. As a supplement, 144 data pairs used by Shen et al. [27] are also included. In total, there are 210 SPM-*R_rs_* data pairs used for the recalibration.

For the valid data pairs, the hyperspectral in situ *R_rs_* spectrum is transformed into *R_rs_* values at each band of WFV cameras via:

(7) $$R_{rsi}=\frac{\int_{\lambda_{min}}^{\lambda_{max}}f_{i}\left(\lambda \right)R_{rs}\left(\lambda \right)d\lambda }{\int_{\lambda_{min}}^{\lambda_{max}}f_{i}\left(\lambda \right)d\lambda },$$

where $R_{rsi}$ is the simulated *R_rs_* value at *i*th band of WFV; $f_{i}\left(\lambda \right)$ is the Spectral Response Function (SRF) of the WFV at *i*th band; $R_{rs}\left(\lambda \right)$ is the in situ measured hyperspectral *R_rs_* spectrum; $\lambda_{max}$ and $\lambda_{min}$ is the upper and lower limit of the spectral range respectively.

**Figure 2:**
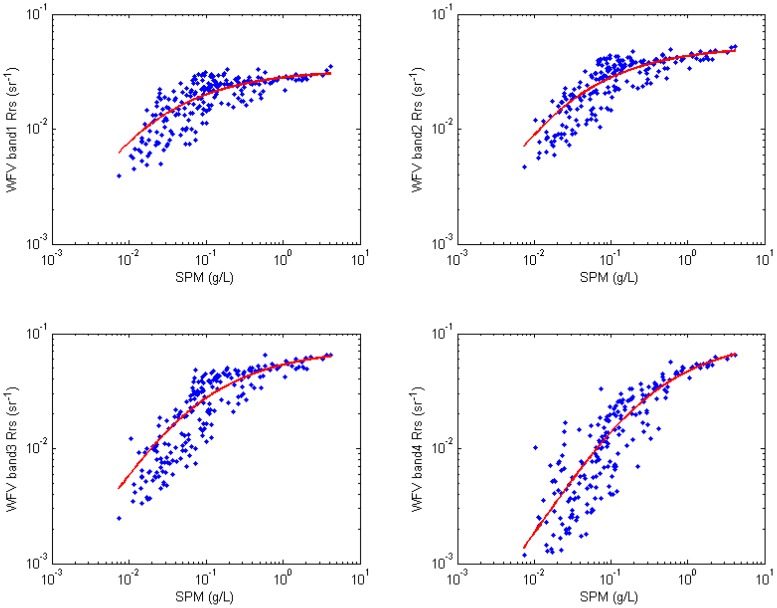
Scatter plots of simulated WFV *R_rs_* at each band (y-axis) and in-situ measured *C*_SPM_ (x-axis), red line is the non-linear regression curve in least-squares sense in the form of Equation (5).

A non-linear curve-fitting problem in least-squares sense was solved for the simulated WFV *R_rs_* at each band and in situ measured *C*_SPM_ to determine *α* and *β* values in Equation (5) of each WFV band. Scatter plots of simulated *R_rs_* at four bands and *C*_SPM_ are shown in Figure 2 and the recalibration result is shown in Table 3.

### 3.3. Atmospheric Correction Scheme

**Figure 3:**
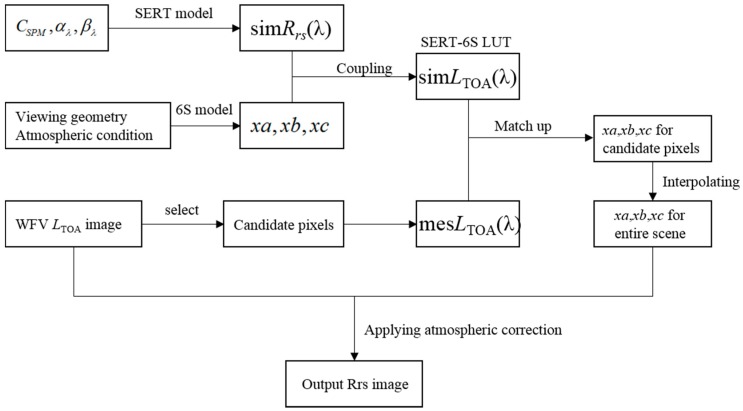
Flow char of the atmospheric correction scheme in this study.

The accuracy of satellite-retrieved SPM concentration depends on the accuracy of the atmospheric correction (i.e., the conversion of the TOA radiance received by satellite-borne sensors to remote sensing reflectance). In this work, we proposed an atmospheric correction method based on a coupled SERT-6S look-up table for GF-1/WFV imagery over turbid waters. Firstly, a look-up table of the WFV *L*_TOA_ at a certain viewing geometry, atmosphere condition and SPM concentration is generated by coupling the 6S atmosphere radiative transfer model and the SERT semi-empirical radiative transfer model. Then a number of candidate water pixels are selected from the WFV *L*_TOA_ image to match up the look-up table, thus there coefficients (*xa*, *xb*, *xc*) of each candidate pixel needed for atmospheric correction can be determined. At last, the atmospheric correction coefficients of the entire scene is acquired by interpolating using the candidate pixels’ and the atmospheric correction procedure can be achieved. Flow chart of this atmospheric correction scheme is shown in Figure 3.

The 6S model is an open source atmosphere radiative transfer model which can be download at http://6s.ltdri.org/, in this study we use the latest version available (6S V2.1) released in June 2015. Inputs of 6S model include viewing geometry, sensor’s spectral response function, atmosphere and aerosol information, etc. Out-puts of the 6S model include three atmospheric correction coefficients (*xa*, *xb*, *xc*). Assuming that the surface is of uniform Lambertian reflectance and the atmosphere is horizontally uniform and various, the reflectance received by the sensor ρ^*^ can be expressed as [31]:

(8) $$\rho^{*}\left(\theta_{s},\theta_{v},\varphi_{s},\varphi_{v}\right)=T_{g}\left(\theta_{s},\theta_{v}\right)\left[\rho_{a}+T\left(\theta_{s}\right)T\left(\theta_{v}\right)\frac{\rho_{ac}}{1-S\times \rho_{ac}}\right],$$

where *θ*_s_ and *θ*_v_ is the sun and viewing zenith angle respectively, *ϕ*_s_ and *ϕ_v_* are the azimuth angles; *ρ_a_* is the intrinsic atmospheric radiance expressed in terms of reflectance; $T_{g}\left(\theta_{s},\theta_{v}\right)$ is the factor represents absorption of the atmosphere; $T\left(\theta_{s}\right)$ is the transmittance from the sun to the ground and $T\left(\theta_{v}\right)$ is the transmittance from ground to the sensor; *ρ_ac_* is the atmospherically corrected reflectance; *S* is the spherical albedo of the atmosphere.

The top-of-atmosphere radiance *L*_TOA_ measured by the sensor is linked with the equivalent reflectance ρ^*^ via:

(9) $$\rho^{*}=\frac{\pi L_{\mathrm{TOA}}}{\mu_{s}E_{s}},$$

where *E*_S_ is the solar flux at the top-of-atmosphere and *μ_s_* is the cosine of the sun zenith angle.

From Equation (8) *ρ_ac_* can be determined as:

(10) $$\rho_{ac}=\frac{{\rho_{ac}}^{\prime }}{1+S\times {\rho_{ac}}^{\prime }},\;{\rho_{ac}}^{\prime }=\left[\frac{\rho^{*}\left(\theta_{s},\theta_{v},\varphi_{s},\varphi_{v}\right)}{T_{g}\left(\theta_{s},\theta_{v}\right)}-\rho_{a}\right]÷\left[T\left(\theta_{s}\right)T\left(\theta_{v}\right)\right]$$

From Equations (9) and (10) it can be derived that:

(11) $$\rho_{ac}=\frac{y}{1+xc\times y},\;y=xa\times L_{\mathrm{TOA}}-xb,$$

with:

(12) $$xa=\frac{\pi }{T_{g}T\left(\theta_{s}\right)T\left(\theta_{v}\right)\mu_{s}E_{s}},\;xb=\frac{\rho_{a}}{T\left(\theta_{s}\right)T\left(\theta_{v}\right)},\;xc=S.$$

Under the Lambertian assumption, the remote sensing reflectance *R_rs_* can be simply expressed as:

(13) $$R_{rs}=\frac{\rho_{ac}}{\pi }$$

Thus if the *R_rs_*(*λ*) is known, then the *L*_TOA_(*λ*) can be calculated by combining Equations (11) and (13) via:

(14) $$L_{\mathrm{TOA}}\left(\lambda \right)=\frac{p+xb\left(\lambda \right)}{xa\left(\lambda \right)},\;p=\frac{\pi R_{rs}\left(\lambda \right)}{1-\pi R_{rs}\left(\lambda \right)\times xc\left(\lambda \right)}.$$

For a certain SPM concentration, the *R_rs_*(*λ*) can be calculated by SERT model via Equation (5). By a combination of Equations (5) and (14), simulated *L*_TOA_(*λ*) for a certain SPM concentration, viewing geometry, atmosphere and aerosol condition can be derived. So the look-up table of *L*_TOA_(*λ*) can be generated by batch-running the 6S model with parameters varied within the possible range coupling with *R_rs_*(*λ*) values calculated by SERT model with varied SPM concentrations. Some input parameters and their value ranges for 6S and SERT model in this study are shown in Table 4. Lacking of ground weather station data, we use two standard atmospheric models (Mid-latitude summer and Mid-latitude winter) and three standard aerosol models (Continental, Maritime and Urban) in the 6S model. Because of the spectral response functions at each band of 4 WFV cameras differed from each other a little, the averaged spectral response function at each band was used to define the spectral conditions when running the 6S code.

For radiometric calibrated WFV *L*_TOA_ images, pixels with band 4 (NIR band) *L*_TOA_ less than 30 Wm^−2^·sr^−1^·μm^−1^ are considered to be water pixels. It takes a lot of time if the match-up process implemented pixel by pixel, instead, we randomly select a number of candidate water pixels (2000 for one scene in this study) to match up with the LUT. The Euclidean distances of one candidate pixel’s *L*_TOA_ vector to the simulated *L*_TOA_ vectors in the LUT are calculated, and the nearest record in the LUT is selected to get the atmospheric correction coefficients (*xa*, *xb*, *xc*) for this pixel. In fact, the searching range of the LUT for a certain pixel can be reduced because the viewing geometry information can be acquired from the metadata file of the image. Assume that the atmosphere and aerosol condition over one scene didn’t change much, pixels with atmospheric correction coefficients differs by one times the standard deviation of the rest candidate pixels are removed. Then the atmospheric correction coefficients of the entire scene can be acquired by interpolating using the candidate pixels’. After that, the atmospheric correction process can be done using Equations (11) and (13) pixel by pixel.

## 4. Results and Discussion

### 4.1. GF-1/WFV Imagery Processing

**Figure 4:**
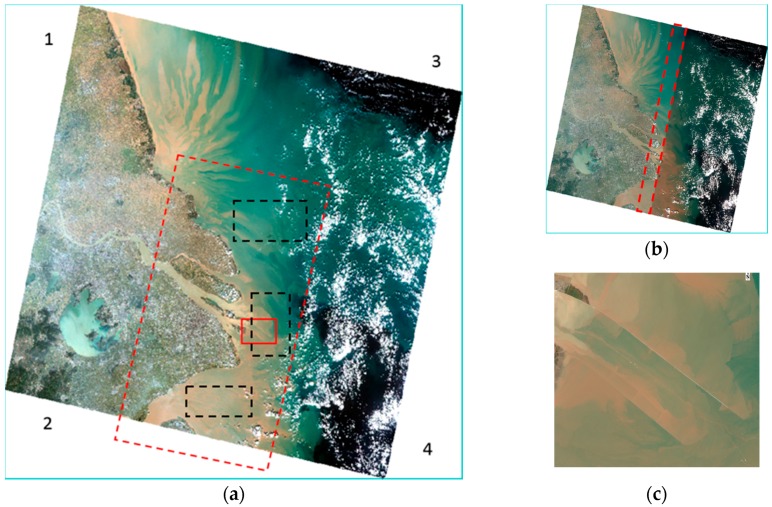
(**a**) Mosaic RGB (channels 3-2-1) image of two WFV1 *L*_TOA_ (marked with number 1 and 2) images and two modified WFV2 *L*_TOA_ (marked with number 3 and 4) images acquired at 14 November 2014. Dashed red box shown the coverage area of two Landsat-8/OLI images acquired at the same day, solid red box shown the boundary of (**c**) and three dashed black boxes indicate the region-of-interests for sampling in following chapters; (**b**) Mosaic RGB image of two WFV1 *L*_TOA_ and two raw WFV2 *L*_TOA_, dashed red box indicates the overlay part of WFV1 and WFV2 images. (**c**) A detail of deep-water channel.

**Figure 5:**
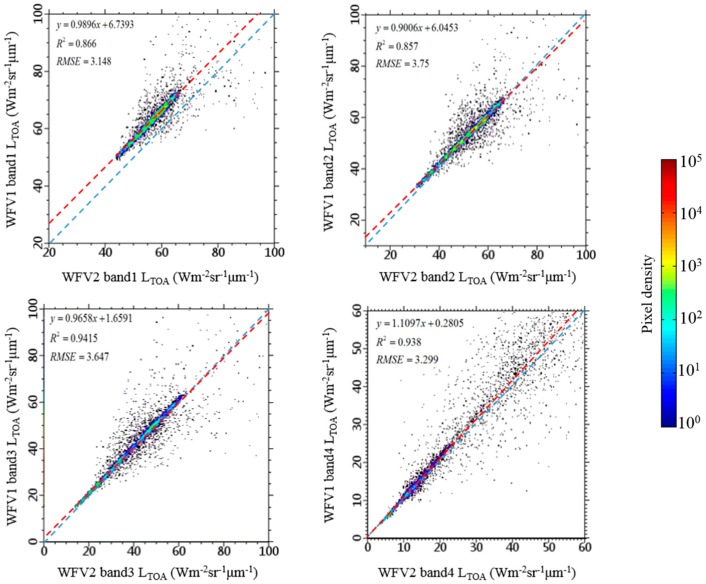
Comparison between WFV1 (y-axis) and WFV2 (x-axis) *L*_TOA_ of corresponding pixels (*N* = 4,640,117) at each band. Pixels are within two cameras’ images overlay part (dashed red box in Figure 4b). Colors denote pixel densities, the dashed red line is the regression line and the dashed blue line is the 1:1 line.

Figure 4a shows the mosaic RGB (channels 3-2-1) image of 4 GF-1/WFV images acquired on 4 November 2014, radiometric calibration was applied before the mosaic process, and the mosaic process is achieved using ENVI (version 5.2) Seamless Mosaic workflow. The mosaic image consists of two WFV1 camera images (marked with number 1 and 2) and two WFV2 camera images (marked with number 3 and 4). The *L*_TOA_ at each band of WFV2 camera images have been modified using the linear fit relationship shown in Figure 5. Figure 4b shows the mosaic of WFV1 *L*_TOA_ images and raw WFV2 *L*_TOA_ images, the chromatism of two cameras’ images can be seen obviously. The detail of deep-water channel located in the North Passage (solid red box in Figure 1a) is shown in Figure 4c. Coverage area of two Landsat-8/OLI images acquired at the same day is also shown in Figure 4a (dashed red box).

Figure 5 shows the comparison of *L*_TOA_ at each bands between WFV1 and WFV2 images’ corresponding pixels within the overlay part (see Figure 4b dashed red box), and a good linear relationship was found. The linear regression line (Figure 5 red dashed line) diverge from the 1:1 line (Figure 5 dashed blue line) mainly because: (1) the viewing geometry of two sensors are different; (2) the inherent difference between two sensors (e.g., the Spectral Response Function of different WFV cameras varied from each other slightly). For this study, the largest divergence between WFV1 *L*_TOA_ and WFV2 *L*_TOA_ occurred at band 1 (blue band), and the difference is about 6.5 Wm^−2^·sr^−1^·μm^−1^ within the data range. Except for band 1, the *L*_TOA_ of WFV1 and WFV2 differed from each other slightly, for most samples the differences are within 2 Wm^−2^·sr^−1^·μm^−1^.

**Figure 6:**
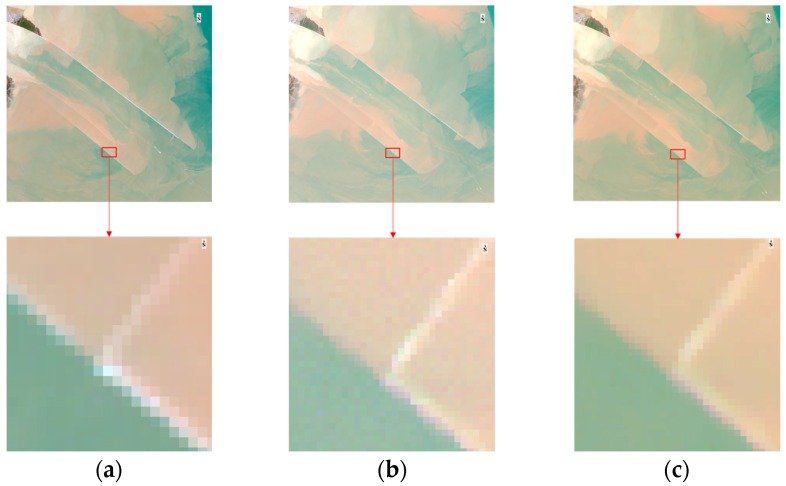
RGB image of: (**a**) Landsat-8/OLI (channels 4-3-2) *L*_TOA_; (**b**) Raw GF-1/WFV (channels 3-2-1) *L*_TOA_; (**c**) GF-1/WFV (channels 3-2-1) *L*_TOA_ after sliding window smooth. Images are acquired at same date (4 November 2014), upper figure is the deep-water channel shown in Figure 4c (red solid box in Figure 4a) and lower figure is the zoom-in of the red box in upper figure.

As discussed in Section 3.1, relatively low signal-to-noise ratio of the WFV cameras may leads to a “noisy” image when zoom in comparing with Landsat-8/OLI image (see Figure 6a,b). After applying the sliding-window filtering the “noisy” problem becomes unobvious (see Figure 6c).

Whether to apply the filtering process must be considered for the reasons that: (1) the filtering process may lead to the loss of spatial detailed information and cause artificial smoothing of edges or fronts; and (2) the filtering process is time-consuming. 

**Figure 7:**
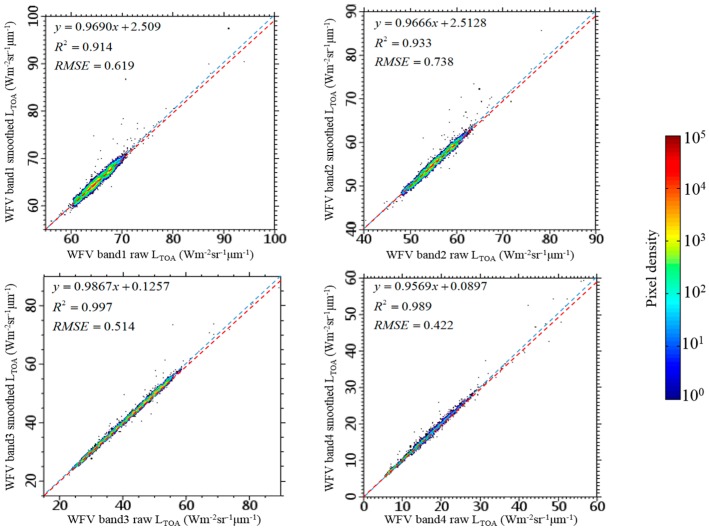
Comparison between raw (x-axis) and smoothed (y-axis) WFV *L*_TOA_ of corresponding pixels (*N* = 4,465,514) at each band. Pixels are within region-of-interests for sampling (three dashed black box in Figure 4a). Colors denote pixel densities, the dashed red line is the regression line and the dashed blue line is the 1:1 line.

Comparison on pixel values of WFV LTOA before and after the filtering process is shown in Figure 7, the pixels are within three sampling regions shown in Figure 4a (dashed black box). The regression line (dashed red line) are slightly differed from the 1:1 line (dashed blue line) for all bands indicates that the WFV LTOA have small changes after the filtering process.

### 4.2. Atmospheric Correction and Intercomparison

Before the atmospheric correction procedure described in Section 3.3, the four scenes of GF-1/WFV imagery acquired at 4 November 2014 were firstly radiometric calibrated using Equation (1) to gain the *L*_TOA_ images. Then the 3 × 3 sliding-window smoothing was applied to all images to reduce the “noisy” phenomenon. After that the WFV2 *L*_TOA_ images were adjusted using the linear regression equation shown in Figure 7. Memory overflow problem appeared when applying the atmospheric correction procedure to the 4-scenes mosaic image even for a large RAM (20 GB for our PC), so we apply the procedure to each scene separately to gain the *R_rs_* value.

Lacking of quality controlled in situ data for the validation of our atmospheric correction method, we use the Landsat-8/OLI imagery acquired at the same date for cross-validation. Two scenes of Landsat-8/OLI imagery at L1T on 4 November 2014 of our study area were processed using ACOLITE (version 20160520.1) with the per pixel variable epsilon SWIR atmospheric correction method [5,10] to gain the *R_rs_* value. The green and red band (band 3 and band 4 for OLI, band 2 and band 3 for WFV) were chosen for comparison. 

**Figure 8:**
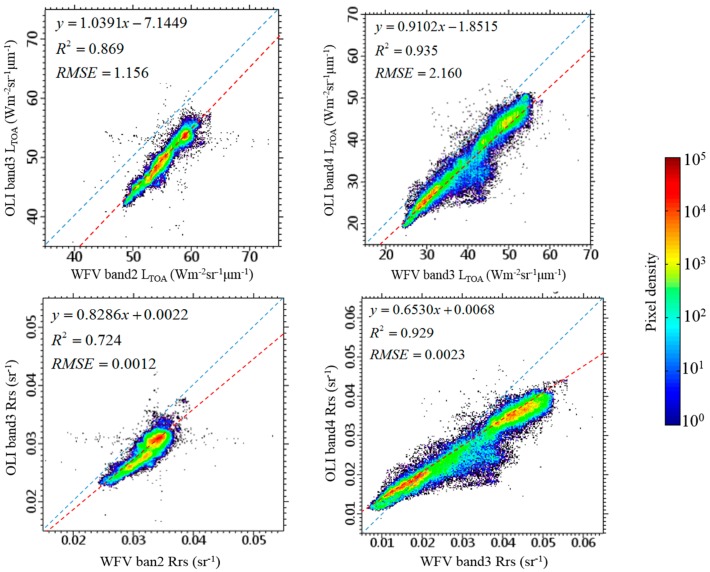
Comparison between WFV’s and OLI’s *L*_TOA_ (upper two figures) together with *R_rs_* (lower two figures) of corresponding pixels (*N* = 4,465,514) at green (band 2 for WFV, band 3 for OLI) and red (band 3 for WFV, band 4 for OLI) band. Pixels are within region-of-interests for sampling (three dashed black box in Figure 4a). Colors denote pixel densities, the dashed red line is the regression line and the dashed blue line is the 1:1 line.

The comparison for *L*_TOA_ and atmospheric correction result (*R_rs_*) of two sensors is shown in Figure 8. It can be seen that the regression lines of WFV-OLI *R_rs_* comparison are closer to the 1:1 line compared to the *L*_TOA_’s, indicated that both atmospheric correction methods can remove part of the differences caused by sensor’s type and viewing geometry. One possible reason lead to the difference between WFV-OLI *R_rs_* regression line and 1:1 line is that there exists about half an hour’s time lag among the two images’ central time. This reason has to be considered especially in a region with large tidal dynamics or for images with high spatial resolution.

**Figure 9:**
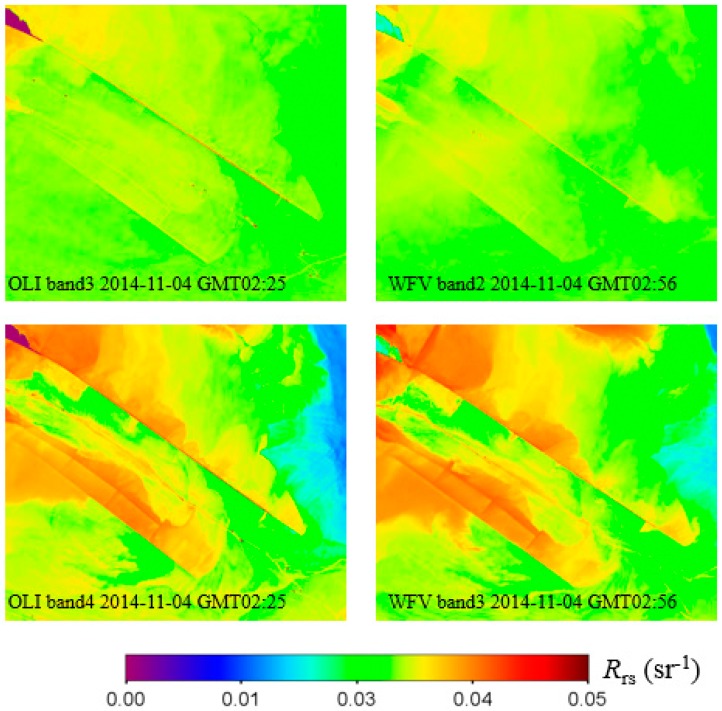
The *R_rs_* image of deep water channel retrieved by OLI image using SWIR method [10] and by WFV image using our method. Upper two figures corresponding to green band and lower two figures to red band.

Figure 9 shows the *R_rs_* images of two sensors’ green and red band (band 2 and band 3 for WFV, band 3 and band 4 for OLI) over the deep-water channel (Figure 4c), and a good correspondence is found both in terms of absolute values and spatial patterns. As shown in Figure 8, the WFV-derived *R_rs_* is a little bit higher than the OLI’s in general. For both sensors, the *R_rs_* image of red band holds more information than the green band’s. 

### 4.3. Quantitative Estimation of Suspended Particulate Matter and Validation

**Figure 10:**
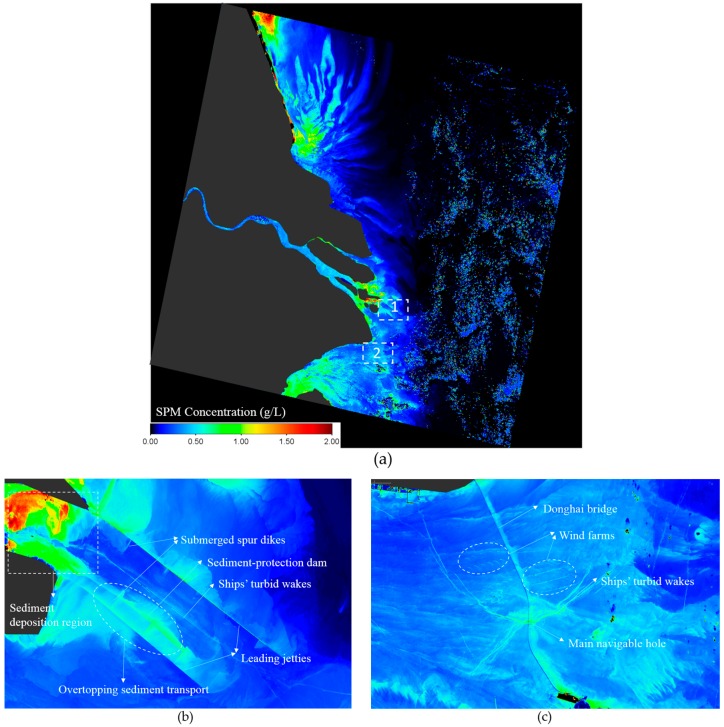
(**a**) The SPM concentration derived by the atmospherically corrected WFV band 3 *R_rs_* image using SERT model over the Yangtze Estuary and its adjacent sea; (**b**) Zoom-in of the deep-water channel (box1 in Figure 10a); (**c**) Zoom-in of the Donghai Bridge (box2 in Figure 10a).

After the atmospheric correction procedure, it’s possible to determine the SPM concentration using the *R_rs_* images. SERT model is chosen for C_SPM_ retrieval, as shown in Figure 9, the *R_rs_* image of WFV band 3 (red band) holds more spatial and quantity information, so the SERT model is applied to the red band of WFV *R_rs_* images to derive the SPM concentration (shown in Figure 10), which is also suggested by Shen et al. [26] for regions with high SPM concentration.

Thanks to high spatial resolution of WFV images, many spatial detailed information of SPM that can hardly be seen by traditional ocean color sensors (e.g., MODIS, MERIS, GOCI and so on) now can be observed using the WFV SPM product. Figure 10b shows the SPM concentration over the deep water channel, the flow and sediment transport overtopping the south leading jetty can be observed, and the highest SPM concentration at the top left of the figure indicates the serious sediment deposition region. Figure 10c shows the SPM concentration over the Donghai Bridge, which is 32.5 km in length and connects mainland Shanghai with the Yangshan Deep-Water Port. The SPM concentration at the eastern side of the bridge is higher than the western side indicates the bridge piers’ block effect of the sediment transport. Off shore wind farms can also be observed, and the merge point of the ships’ turbid wakes is the main navigable hole.

**Figure 11:**
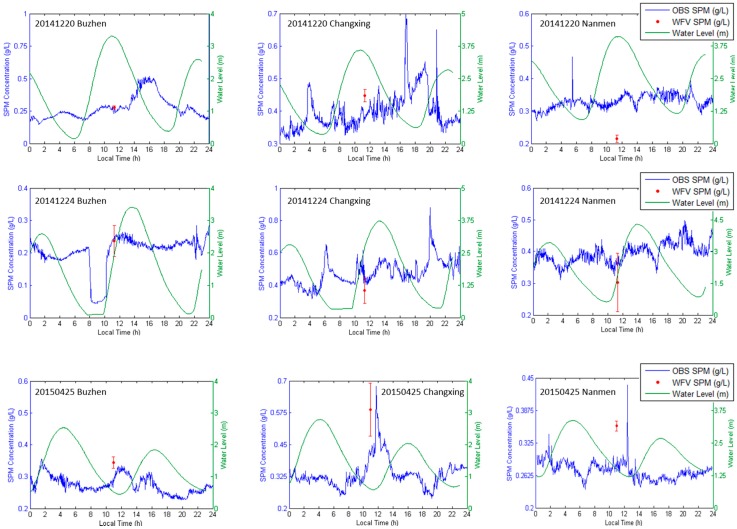
Comparison between WFV derived SPM and fixed-station OBS observed SPM at three fixed station (Nanmen, Changxing and Buzhen) on three days (20 December 2014, 24 December 2014 and 25 April 2015). Red point is the average of WFV derived SPM concentrations of pixels within a 3 × 3 box centered at the station’s location, and the error bar indicated the standard deviation (STD). Green line and blue line is the OBS observed water level and SPM concentration respectively.

In order to assess the accuracy of the SPM products, observations of three fixed-stations (Nanmen, Buzhen and Changxing, red stars in Figure 1) were selected for the inter-comparison with the satellite-derived SPM. Three cloud-free scenes of WFV image on 20 December 2014, 24 December 2014 and 25 April 2015 were selected for the comparison, and the SPM concentration is derived by the SERT model using WFV band 3 *R_rs_* images. The satellite-derived SPM concentration of each fixed-station is an average of SPM concentrations of pixels within a 3 × 3 box centered at the station’s location. The fixed-station SPM concentration is transformed from the turbidity observed by the D&A Tech optical backscatter instrument (OBS) with the linear relationship developed by Xue, He and Wang [32]:

(15) y=0.0017x+0.0202,

where x is the OBS-observed turbidity in unit of NTU and y is the SPM concentration in unit of g/L. The result is shown in Figure 11.

It can be seen from Figure 11 that the OBS-derived SPM is close to the WFV-derived for most occasions, especially for Buzhen station, the differences between the SPM concentrations are less than 0.02 g/L for validation days. The Nanmen station has the largest variance, up to 0.08 g/L on 20 December 2014 and 0.06 g/L on 25 April 2015. One possible reason that may cause the variance is that the OBS instruments of different stations may have different relationships when convert turbidity to SPM concentration, so a unique relationship may cause the differences. Besides, the performance of the OBS instruments may change with time, so the linear relationship may also changes.

## 5. Conclusions

Having advantages of high spatial resolution, wide swath width and relatively short revisit time making the GF-1/WFV imagery an ideal tool for costal and estuarine waters quality monitoring. In this work we proposed an atmospheric correction scheme for the GF-1/WFV imagery over turbid waters. The atmospheric correction scheme is based on a look-up table coupling SERT and 6S models. 210 in situ SPM-*R_rs_* data pairs are used for the recalibration of SERT model to acquire the *α* and *β* values of each WFV band, and the 6S model is batch run with varied input parameters. The WFV derived *R_rs_* using our method is compared with the Landsat-8/OLI derived *R_rs_* using per pixel variable epsilon SWIR atmospheric correction method [5,10] and good correlation was found both in absolute values and spatial pattern.

Thanks to its high spatial resolution, the WFV SPM product shows many detail information about the influence of human’s activity and artificial engineering on SPM distribution, which is very important for estuarine and coastal waters where many human activities take place. In order to assess the WFV SPM product, the fixed station observations were used and the results are acceptable.

## Figures and Tables

**Table 1:** Comparison of band range, signal-to-noise ratio (SNR) [15] and extraterrestrial solar irradiance (*F*_0_) between Landsat-8/OLI and GF-1/WFV.

Band ID	Band Range (nm)		SNR		*F*_0_ (W m^−2^·μm^−1^)	
	GF-1/WFV	L-8/OLI	GF-1/WFV	L-8/OLI	GF-1/WFV	L-8/OLI
1 (Coastal/aerosol)	-	433–453	-	-	-	1895.6
2 (Blue)	450–520	450–515	78.127	122.798	1966.8	2004.6
3 (Green)	520–590	525–600	52.672	96.358	1822.6	1820.7
4 (Red)	630–690	630–680	40.815	111.941	1523.2	1549.4
5 (NIR)	770–890	845–885	34.610	92.315	1066.5	951.2
6 (SWIR1)	-	1560–1660	-	-	-	247.6
7 (SWIR2)	-	2100–2300	-	-	-	85.5
8 (PAN)	-	500–680	-	-	-	1724.0
9 (CIRRUS)	-	1360–1390	-	-	-	367.0

**Table 2:** The sensor ID (of GF-1/WFV), scene center latitude/longitude (in degree) and scene center time (Greenwich Mean Time) of images used in this study.

Aquired Date	GF-1/WFV				Landsat-8/OLI		
	Sensor	Center Lat (°)	Center Lon (°)	Center Time (GMT)	Center Lat (°)	Center Lon (°)	Center Time (GMT)
4 November 2014	WFV1	N31.3	E120.8	02:56:54	N31.6	E121.9	02:25:13
	WFV1	N33.0	E121.2	02:56:26			
	WFV2	N31.0	E122.9	02:56:54	N30.3	E121.5	02:25:36
	WFV2	N32.6	E123.3	02:56:26			
20 December 2014	WFV4	N31.8	E121.8	03:19:16			
24 December 2014	WFV4	N31.9	E121.8	03:17:04			
25 April 2015	WFV1	N31.3	E121.7	02:56:36			

**Table 3:** *α* and *β* values of WFV camera at each band, derived from the non-linear regression shown in Figure 2. The absolute percentage difference (APD), root mean square error (RMSE), and determination coefficient (*R*^2^) of the regression at each band is also listed.

Sensor	Band ID	*α*	*β*	APD (%)	RMSE (sr^−1^)	*R*^2^ (%)
WFV	Band 1 (blue)	0.0329	78.33	26.7	0.0048	60.7
WFV	Band 2 (green)	0.0530	47.94	27.6	0.0067	70.8
WFV	Band 3 (red)	0.0746	18.32	42.4	0.0082	77.9
WFV	Band 4 (NIR)	0.0935	4.066	53.1	0.0058	87.2

**Table 4:** Input parameters for 6S and SERT model and their varied range in this study.

Model	Parameter Name	Data Range	Step
6S	Solar zenith angle	0°~70°	5°
	Sensor zenith angle	0°~70°	5°
	Scattering angle	0°~180°	45°
	Scene aquired date (Julian day)	1~365	60
	Atmospheric model	Midlatitude summer	-
		Midlatitude winter	
	Aerosol type	Continental	-
		Maritime	
		Urban	
	Aerosol optical depth at 550 nm	0.05–2.00	0.05
	WFV band	1–4	1
SERT	SPM concentration	0.001 g/L~10 g/L (N = 100 in log-scale)	

## References

[1] Schoellhamer D.H., Mumley T.E., Leatherbarrow J.E. (2007). Suspended sediment and sediment-associated contaminants in San Francisco Bay. Environ. Res..

[2] El-Asmar H.M., White K. (2002). Changes in coastal sediment transport processes due to construction of New Damietta Harbour, Nile Delta, Egypt. Coast. Eng..

[3] Chen S., Zhang G., Yang S. (2003). Temporal and spatial changes of suspended sediment concentration and resuspension in the Yangtze River estuary. J. Geogr. Sci..

[4] Vanhellemont Q., Neukermans G., Ruddick K. (2014). Synergy between polar-orbiting and geostationary sensors: Remote sensing of the ocean at high spatial and high temporal resolution. Remote Sens. Environ..

[5] Vanhellemont Q., Ruddick K. (2014). Turbid wakes associated with offshore wind turbines observed with Landsat 8. Remote Sens. Environ..

[6] IOCCG Atmospheric Correction for Remotely-Sensed Ocean-Colour Products. http://www.star.nesdis.noaa.gov/sod/mecb/color/documents/IOCCG_report_10.pdf.

[7] Gordon H.R., Wang M. (1994). Retrieval of water-leaving radiance and aerosol optical thickness over the oceans with SeaWiFS: A preliminary algorithm. Appl. Opt..

[8] Ruddick K., Ovidio F., Rijkeboer M. (2000). Atmospheric correction of SeaWiFS imagery for turbid coastal and inland waters. Appl. Opt..

[9] Wang M., Wei S. (2005). Estimation of ocean contribution at the MODIS near-infrared wavelengths along the east coast of the US: Two case studies. Geophys. Res. Lett..

[10] Vanhellemont Q., Ruddick K. (2015). Advantages of high quality SWIR bands for ocean colour processing: Examples from Landsat-8. Remote Sens. Environ..

[11] Li Z.F. (2015). Application of GF-1 Satellite in Land-use Remote Sensing Monitoring. Land Resour. Herald.

[12] Wu P.Q., Zhang J., Ma Y. (2015). Positioning Precision Evaluation of GF-1 in the Coastal Zone. Hydrogr. Surv. Chart..

[13] Zhang K., Ma S.B., Li Z.R., Liu S.Y. (2016). Geological Interpretation of GF-1 Satellite Imagery. Remote Sens. Inf..

[14] Li F.L., Wang L., Liu J., Chang Q.R. (2015). Remote Sensing Estimation of SPAD Value for Wheat Leaf Based on GF-1 Data. Trans. Chin. Soc. Agric. Mach..

[15] Liang W.X., Li J.S., Zhou D.M., Shen Q., Zhang F.F. (2015). Evaluation of GF-1 WFV Characteristics in Monitoring Inland Water Environment. Remote Sens. Technol. Appl..

[16] Cheng Q., Liu B., Li T., Zhu L. (2015). Research on remote sensing retrieval of suspended sediment concentration in Hangzhou Bay by GF-1 satellite. Mar. Environ. Sci..

[17] Zhu L., Li Y.M., Zhao S.H., Guo Y.L. (2015). Remote sensing monitoring of Taihu Lake water quality by using GF-1 WFV data. Remote Sens. Land Resour..

[18] He Q., Yun C.X., Shi W.R. (1999). Remote sensing analysis for suspended sediment concentration in water surface layer in Yangtze River estuary. Prog. Nat. Sci..

[19] Bai Z.G. (2013). The technical feature of GF-1 satellite. Aerosp. China.

[20] Irons J.R., Dwyer J.L., Barsi J.A. (2012). The next Landsat satellite: The Landsat Data Continuity Mission. Remote Sens. Environ..

[21] Zhu B., Wang X.H., Tang L.L., Li C.R. (2010). Review on Methods for SNR Estimation of Optical Remote Sensing Imagery. Remote Sens. Technol. Appl..

[22] CRESDA Absolute Radiometric Calibration Coefficient. http://www.cresda.com/CN/Downloads/dbcs/6709.shtml.

[23] RBINS ACOLITE. http://odnature.naturalsciences.be/remsem/software-and-data/acolite.

[24] Mueller J.L., Fargion G.S., Mcclain C.R., Pietras C., Hooker S.B., Austin R.W., Miller M., Knobelspiesse K.D., Frouin R., Holben B. Ocean Optics Protocols For Satellite Ocean Color Sensor Validation, Revision 4, Volume II: Instrument Specifications, Characterization and Calibration. https://ntrs.nasa.gov/archive/nasa/casi.ntrs.nasa.gov/20020044096.pdf.

[25] Leonid G.S., Shen F. (2014). Optical closure for remote-sensing reflectance based on accurate radiative transfer approximations: The case of the Changjiang (Yangtze) River Estuary and its adjacent coastal area, China. Int. J. Remote Sens..

[26] Shen F., Verhoef W., Zhou Y., Salama M.S., Liu X. (2010). Satellite Estimates of Wide-Range Suspended Sediment Concentrations in Changjiang (Yangtze) Estuary Using MERIS Data. Estuar. Coasts.

[27] Shen F., Zhou Y., Peng X., Chen Y. (2014). Satellite multi-sensor mapping of suspended particulate matter in turbid estuarine and coastal ocean, China. Int. J. Remote Sens..

[28] Kubelka P., Munk F. (1931). Ein beitrag zur optik der farbanstriche. Z. Tech. Phys..

[29] Shen F., Zhou Y., Li J., He Q., Verhoef W. (2013). Remotely sensed variability of the suspended sediment concentration and its response to decreased river discharge in the Yangtze estuary and adjacent coast. Cont. Shelf Res..

[30] Gordon H.R., Brown O.B., Jacobs M.M. (1975). Computed Relationships between the Inherent and Apparent Optical Properties of a Flat Homogeneous Ocean. Appl. Opt..

[31] Vermote E.F., Tanre D., Deuze J.L., Herman M., Morcrette J.J. Second simulation of a satellite signal in the solar spectrum-vector (6SV). Proceedings of the Geoscience and Remote Sensing Symposium, 1990 (IGARSS’90) ‘Remote Sensing Science for the Nineties’, 10th Annual International.

[32] Xue Y.Z., He Q., Wang Y.Y. (2004). The method and application of OBS in the measurement of sediment concentration. J. Sediment Res..

